# The culture of research communication in neonatal intensive care units: key stakeholder perspectives

**DOI:** 10.1038/s41372-021-01221-4

**Published:** 2021-10-26

**Authors:** Jennifer Degl, Deb Discenza, Yamile Jackson, Keira Sorrells, International Neonatal Consortium

**Affiliations:** 1 Speaking for Moms & Babies, Inc., Mahopac, NY USA; 2 Preemie World, Springfield, VA USA; 3 Nurtured by Design, Sugar Land, TX USA; 4 NICU Parent Network, Madison, MS USA

**Keywords:** Paediatrics, Medical research

In “*The Culture of Research Communication in Neonatal Intensive Care Units: Key Stakeholder Perspectives”* published in the *Journal of Perinatology*, we are reminded of the differences in perception of various aspects of neonatal research that exist between key stakeholders who are integral to the care of premature and medically fragile babies in the neonatal intensive care unit (NICU) [1]. The Communications Workgroup of the *International Neonatal Consortium (INC)* surveyed three key stakeholder groups including neonatologists, neonatal nurses, and parents who had infants cared for in a NICU. The goal of these parallel stakeholder surveys was to assess personal perspectives on research-related communication and education practices in multiple NICUs and health care systems around the world. The survey evaluated perspectives on the role for research in the NICU, the current communication flow in the NICU, education and training of neonatal personnel on the role of neonatal research, the research consent process, and disclosure of study results to families. By analyzing data from the surveys, it was clear that there are significant differences in perception regarding communication flow and research practices in NICUs within these three key stakeholder groups.

These results were not unexpected. Although variations in perceptions of care and care practice are ubiquitous across most fields of medicine, the results of the surveys led us to focus on a few key messages that we (as NICU parents) believe are important to share. While family-centered care is becoming more prevalent, 20% of parents indicated that they were never or rarely offered meetings with the neonatal team caring for their baby. This raises serious concerns for NICU parents as well as other key stakeholders. recommend timely multidisciplinary family meetings for all critically ill patients, including babies, since these meetings are well recognized to promote shared decision-making [2]. While rounds provide parents a great way to learn critical information about their baby, some parents cannot be present during rounds and even those who can attend may be confused by the medical jargon used. Ideally, parents should be offered regular meetings with key members of their baby’s healthcare team, where they are encouraged to ask questions and offer information. We hope that the improved communication strategies and research practices in NICUs that we recommend will ultimately benefit future babies and their families.

Healthcare provider and parent perceptions differ regarding the inclusion of NICU graduate parents in research education and training programs, with 70% of parents reporting that they did not participate in any such programs prior to discharge. Graduate parents have a wealth of information to offer members of the neonatal team and to parents who have babies being cared for in the NICU. Their experience should not be underrated or understated. We believe that NICU graduate parents should regularly meet with neonatal physicians and nurses to provide their perspective on everything from care practices to how to conduct conversations and communicate with parents in a trauma-informed [3] manner. Graduate parents should also meet with current NICU parents as mentors to help provide peer-to-peer emotional support that only a former NICU parent can.

An overwhelming proportion of physicians do not feel that existing medications are sufficient to meet the medical needs of sick babies. This presents an excellent opportunity for physicians to demand that pharmaceutical companies and government agencies work on developing medications exclusively for premature babies so that current medications do not have to be used off-label in unstudied dosages to save babies (as is often the case). All stakeholders seem to embrace the concept of research as core to the NICU mission and research as an essential component to improving neonatal outcomes. There is a prevalent misconception that parents do not want their babies to participate in research and yet the survey showed the opposite to be true. Knowing that parents do in fact see the importance of neonatal clinical research, further investigation needs to examine why more babies are not enrolled in research studies and what can be done to improve the enrollment process.

Only a small proportion of professional respondents noted that consent for study participation is *‘always’* or *‘usually’* sought from families before the birth of a sick baby or in advance of an anticipated event. We see a clear opportunity for improvement regarding the timing of informed consent, as well as the information presented to parents before they are asked for consent. Parents at risk for delivering a preterm baby should be informed of the possibility that they may be asked to enroll their baby in a clinical study, preferably before a premature or emergent delivery occurs. When a baby is born preterm or under emergent circumstances, parents are often bombarded with a great deal of information and asked to make critical decisions while under extreme duress. If parents can be educated and informed earlier in the process, this allows them the time they need to process the information and ask appropriate questions with a clear mind.

One-third of the parents who provided consent for trial participation did not feel it was clear where they could obtain information about study timelines. While there was agreement from all stakeholders that results should be made available to families upon study completion, only one-third of professional respondents noted that their institution has a standard approach in place to provide study results to parents. Among the neonatologists and nurses, there was significant variability (60% vs 35%, respectively) noted on whether their institution has a standard approach in place to maintain up-to-date contact information for parents after study completion. Furthermore, we were concerned that the majority of parents who consented for their baby’s trial participation were not informed/advised about the plan for the disclosure of results, nor was their preference sought on the method of disclosure.

It was clear that parents of babies who consented to participation in clinical trials wanted to be informed of the results. Complicating this issue were the survey findings that identified acknowledgements by neonatologists and nurses that their institutions lack a standard approach to disclosure of study results. We believe this finding should be communicated to all stakeholders and efforts should be made to develop procedures that ensure that parents are informed of study results. The research team should recognize that they are being compensated for their work while the families are being asked to provide their child’s clinical information, biologic specimen(s), and outcomes data while potentially putting their baby at risk by participating in research. The desire to know how the study concluded and if their baby’s participation somehow contributed to the greater good is more than a reasonable request. Parents should be asked if they want to receive trial results and if they do, they should be asked about the best method of communication for them to receive the results. Additionally, a standardized procedure should be in place that allows parents to update their contact information should it change before the conclusion of the study.

## Opportunities from the parent perspective

Many lessons can be learned from this study, but at the very least we feel that these results should encourage healthy conversations between the neonatal clinical and research communities indicating that research should be a core mission of every NICU. Once that mission is adopted, the next steps would be to collaborate on: (1) developing better communication and education regarding how neonatal research is operationalized, (2) how this mindset creates benefits for patients and families, and (3) how parents can help achieve these aims. For successful neonatal research, all NICU stakeholders must have a clear understanding of the various purposes of research (drug trials versus academic studies) and parents need to have a clear understanding of the processes in place to safeguard their babies from harm (which builds trust).

We must never forget that each research subject is someone’s child - a living and breathing baby that was born prematurely or with significant medical issues. The primary goal is to always give babies their best start in life and help them overcome any obstacles that may occur. Instead of viewing these survey results through the lens of what has not been done correctly, we must use the information we received as an opportunity to help babies and families by improving the NICU research environment. Surveys like this can be used to reform research practice and policy and encourage better communication between all the stakeholders most closely involved in the care of babies. This will not only help to improve the quality of clinical studies but will also improve the outcome for each premature and medically fragile baby as well as the NICU experience of the entire family.

## Supplementary information

INC Consortia Members
